# Loss of GATA4 and GATA6 Expression Specifies Ovarian Cancer Histological Subtypes and Precedes Neoplastic Transformation of Ovarian Surface Epithelia

**DOI:** 10.1371/journal.pone.0006454

**Published:** 2009-07-31

**Authors:** Kathy Qi Cai, Corrado Caslini, Callinice D. Capo-chichi, Carolyn Slater, Elizabeth R. Smith, Hong Wu, Andres J. Klein-Szanto, Andrew K. Godwin, Xiang-Xi Xu

**Affiliations:** 1 Sylvester Comprehensive Cancer Center, Department of Medicine, and Department of Obstetrics and Gynecology, University of Miami School of Medicine, Miami, Florida, United States of America; 2 Ovarian Cancer Program, Fox Chase Cancer Center, Philadelphia, Pennsylvania, United States of America; 3 Department of Pathology, Fox Chase Cancer Center, Philadelphia, Pennsylvania, United States of America; Cincinnati Children's Research Foundation, United States of America

## Abstract

**Background:**

The family of zinc finger-containing GATA transcription factors plays critical roles in cell lineage specification during early embryonic development and organ formation. GATA4 and GATA6 were found to be frequently lost in ovarian cancer, and the loss is proposed to account for dedifferentiation of the cancer cells.

**Methodology/Principal Findings:**

We further investigated the expression of GATA4 and GATA6 in ovarian surface epithelial lesions and histological subtypes of ovarian carcinomas by immunostaining. GATA4 and GATA6 were found to be absent in high percentages (80 to 90%) of serous, clear cell, and endometrioid ovarian cancer examined. In contrast, both were found positive in 11 out of 12 cases of mucinous carcinomas, suggesting the expression of the GATA factors can distinguish mucinous cancer from other histological subtypes. GATA4 was frequently lost in preneoplastic lesions such as morphologically normal inclusion cysts and epithelial hyperplasia adjacent to malignant cells. The loss of GATA6 correlates closely with neoplastic morphological transformation of ovarian surface epithelia. In culture, GATA4 expression was progressively reduced upon passaging primary ovarian surface epithelial cells, which correlated with changes in histone modification of the GATA4 locus. A reduced GATA6 gene dosage as in GATA6 (+/−) mice led to an increased pre-neoplastic changes and inclusion cysts in the ovaries, suggesting the loss of GATA6 contributes to ovarian cancer development.

**Conclusions/Significance:**

This study suggests that the expression status of GATA4 and GATA6 may dictate distinct pathologic pathways leading to serous or mucinous ovarian carcinomas. The readily loss of GATA4 expression through changes in chromatin conformation suggests a potential non-phenotypic initiating event, leading to subsequent loss of GATA6, morphological transformation, and ultimate tumorigenesis.

## Introduction

Ovarian epithelial cancer is generally thought to arise from the coelomic epithelium covering the ovarian surface [1], [2] or from inclusion cysts that are also thought to derive from the ovarian surface epithelium [3], [4]. However, there are also strong arguments that at the least some of the ovarian epithelial cancer originated from cells other than of the surface epithelia [5], [6], [7]. Several distinct histological subtypes can be classified based on cell morphology: these are serous, mucinous, clear cell, and endometroid subtypes [8], [9]. One idea to explain the diverse histological subtypes is that ovarian surface epithelial cells undergo metaplasia to adopt various histological characteristics during transformation [2], [8]. Another idea is that ovarian cancer may arise from the remains of mullerian duct structures [6], which are precursors of the fallopian tubes, cervix, uterus, etc, and may present as all the histological subtypes in tumors. Indeed, recent observations lead to the hypothesis that a significant portion of serous ovarian carcinomas may originate from fallopian tubes [5], [7], which have serous like epithelial cells.

Although some characteristics of the precursor cells may retain, the distinction of cancer cells from precursor cells is apparent, and several hallmarks of neoplastic cells have been recognized [10]. Unique genetic mutations are the basis for the neoplastic phenotypes [11]–[13], and additionally cancer cells often appear in an inappropriately differentiated stage, and the term “dedifferentiation” is often used [14]–[16]. The phenotype described as “undifferentiated” or “dedifferentiated” of ovarian cancer often refers to the apparent changes of the cancer cells towards a less epithelial-like morphology or drift from the precursor cells. To provide a molecular interpretation of “dedifferentiation” of cancer cells, one may consider the concept of cell lineage differentiation prominent in developmental biology, in which a primordial cell undergoes changes in its global gene expression profile toward a specific mature cell type. By analogy, dedifferentiation of cancer cells may be caused by the loss of one or more critical genes that are key in inducing the differentiation of a primordial cell type to yield the ovarian surface epithelial cells. Recent studies suggest the loss of the transcription factor GATA6 and GATA4 in ovarian cancer may account for the loss of epithelial characteristics and may be the underlying mechanism of “dedifferentiation” of ovarian cancer cells [17], [18].

The GATA transcription factors bind a consensus A/T-G-A-T-A-A/G sequence in promoters and are conserved in insects and vertebrates, from fly to humans [19]. GATA4 and GATA6 are expressed in most organs, and play critical roles in the development of these organs [20], [21]. GATA4 and GATA6 are first expressed during the formation of extraembryonic endoderm differentiated from the pluripotent embryonic stem cells of the inner cell mass during early embryonic development [22]–[25]. GATA factors are not tissue-specific but rather function in the specification and differentiation of cell lineages within an organ, such as the differentiation of an epithelial cell lineage from stromal cells. GATA4 and GATA6 are expressed in human ovarian surface epithelial cells [17], [18], and presumably, GATA4 and GATA6 are important for the formation and maintenance of the differentiated state of ovarian surface epithelial cells. GATA4 and GATA6 are often lost in ovarian cancer cells [17], [26], and it is speculated that the loss of these developmentally important transcription factors may be the underlying mechanism of dedifferentiation [17], [18].

Whether GATA6 is important for the formation of the ovarian surface epithelium is unknown, since little is known about the derivation of the ovarian surface epithelial cells. Both GATA4 and GATA6 proteins are expressed in ovarian surface epithelial cells but absent in most ovarian cancer cells [17], [18], [26]. We found by ChIP assay that the histone H3 and H4 acetylation of the GATA4 locus decreased greatly in cancer cells comparing to GATA4-positive non-tumor cells [18]. In contrast, histone H3 and H4 acetylation of the GATA6 locus in many cancer cells was not reduced and traces of GATA6 mRNA could be detected by RT-PCR, indicating that GATA6 gene is not transcriptionally silenced but the message is suppressed by other mechanism(s) [18].

Few transcription targets for the GATA factors have been identified, and Disabled-2 (Dab2) is a known transcription target of GATA6 [27]. Dab2 was also found lost in ovarian cancer [28], correlating with the absence of GATA6 and morphological transformation [18]. Dab2 deficiency in mice leads to early embryonic lethality [29]–[31], and heterozygous Dab2 mutant mice develop pre-neoplastic lesions from ovarian surface epithelia [32]. Dab2 is an adaptor protein involved in endocytic trafficking, and Dab2 plays a role in establishing epithelial polarity and surface positioning [33]. Loss of Dab2 is thought to cause morphological transformation of ovarian surface epithelia [34], [35]. Thus, a speculation is that loss of GATA6 will lead to loss of Dab2 and neoplastic morphological transformation of ovarian surface epithelia.

In the current study, we further analyzed the details of GATA4 and GATA6 expression in ovarian tissues and cancer, and examined the impact of the reduction of GATA6 in mice on ovarian surface epithelial transformation.

## Results

### Expression of GATA4 and GATA6 in ovarian cancer histological subtypes

Previously, both GATA4 and GATA6 were observed to be strongly expressed in primary ovarian surface epithelial cells but absent in cultured ovarian cancer cells by both Western blot to examine the protein or Northern blot to measure mRNA [17], [18].

**Figure 1:**
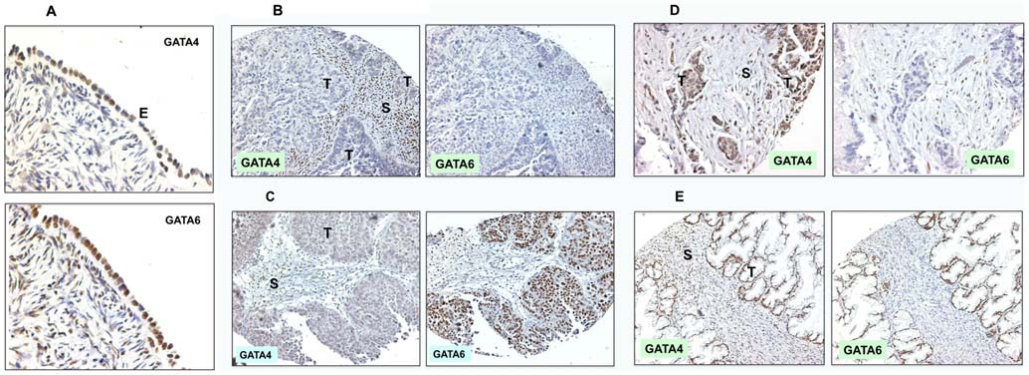
Immunostaining of GATA4 and GATA6 in ovarian cancer histological subtypes. Immunostainings of GATA4 and GATA6 were performed in 111 ovarian carcinomas including the 4 histological subtypes. The results are summarized in Table 1. A. Normal ovarian surface epithelial cells are positive for both GATA4 and GATA6 in the nucleus. 200× magnification. B. An example of both GATA4- and GATA6-negative serous carcinoma. 40× magnification. C. An example of GATA4-negative and GATA6-positive serous carcinoma. 40× magnification. D. An example of GATA4-positive and GATA6-negative serous carcinoma. 40× magnification. E. An example of both GATA4- and GATA6-positive mucinous carcinoma. 40× magnification. E, epithelium; T, tumor; S, stroma.

With further development of antibodies and refinement of staining procedure, we carried out immunohistochemical analysis for the expression of GATA4 and GATA6 in a panel of ovarian non-neoplastic and cancer tissues in tissue microarray (Table 1). Additional 26 non-cancer ovarian tissues from prophylactic oophorectomies were also used in this study. These ovarian tissues were obtained from both high and normal risk women, but no microscopic cancer was found [36]. The 111 informative ovarian carcinomas in tissue microarray used in this study consist of 82 serous, 12 mucinous, 12 clear cell, and 5 endometrioid subtypes (Table 1). In the normal monolayer ovarian surface epithelia, all ovarian surface epithelial cells were stained positive for nuclear GATA4 and GATA6 (Figure 1A). While GATA4 and GATA6 staining are absent or greatly reduced in most ovarian carcinomas (Table 1). Among the informative cases, loss of GATA4 was found in 81/82 (99%) of serous carcinomas, 1/12 (8.3%) of mucinous carcinomas, 11/12 (92%) of clear cell carcinomas, and 5/5 (100%) of endometroid carcinomas. Loss of GATA6 was found in 55/82 (67%) of serous carcinomas, 1/12 (8.3%) of mucinous carcinomas, 10/12 (83%) of clear cell carcinoma, and 5/5 (100%) of endometroid carcinomas. Examples of the immunostainings are shown in Figure 1, for a GATA4- and GATA6-negative serous carcinoma (Figure 1B), a GATA4-negative but GATA6-positive serous carcinoma (Figure 1C), a GATA6-negative but GATA4-positive serous carcinoma (Figure 1D), and a GATA4 and GATA6-positive mucinous carcinoma (Figure 1E). It is also noticeable that some GATA4 and/or GATA6 staining is present in stromal cells in both normal and timorous ovaries, though most of these staining are not as intense as those of the epithelial cells. In some cases as seen in the tumor shown in Figure 1B, some small stromal cells, likely immune and lymphocytic cells, show strong nuclear staining of GATA4.

**Table 1:** Expression of GATA4 and GATA6 in ovarian cancer histological subtypes.

Histological Subtypes	GATA4		GATA6	
	Negative	Positive	Negative	Positive
**Serous**	81/82 (99%)	1/82 (1.2%)	55/82 (67%)	27/82 (33%)
**Mucinous**	1/12 (8.3%)	11/12 (92%)	1/12 (8.3%)	11/12 (92%)
**Clear Cell**	11/12 (92%)	1/12 (8.3%)	10/12 (83%)	2/12 (17%)
**Endometrioid**	5/5 (100%)	0/5 (0%)	5/5 (100%)	0/5 (0%)

GATA4 and GATA6 immunostaining in 111 cases of ovarian carcinomas in TMA..

One striking feature of GATA4 and GATA6 staining is that though GATA4 and GATA6 are lost in most ovarian cancer (predominantly serous subtype), carcinomas of the mucinous subtype are mostly GATA4- and GATA6-positive (Table 1). Thus, the expression of GATA4 and GATA6 distinguishes mucinous carcinomas from other histological subtypes.

### Loss of GATA4 precedes loss of GATA6 in pre-neoplastic ovarian surface and cyst epithelia

GATA4 expression is absent in the majority of serous ovarian cancer, and GATA6 is also absent in many but also positive in some cases (Table 1). Negative GATA6 and positive GATA4 exist only in rare cases of ovarian carcinomas. Thus, loss of GATA4 appears to be more prevalent than loss of GATA6 in ovarian cancer.

**Figure 2:**
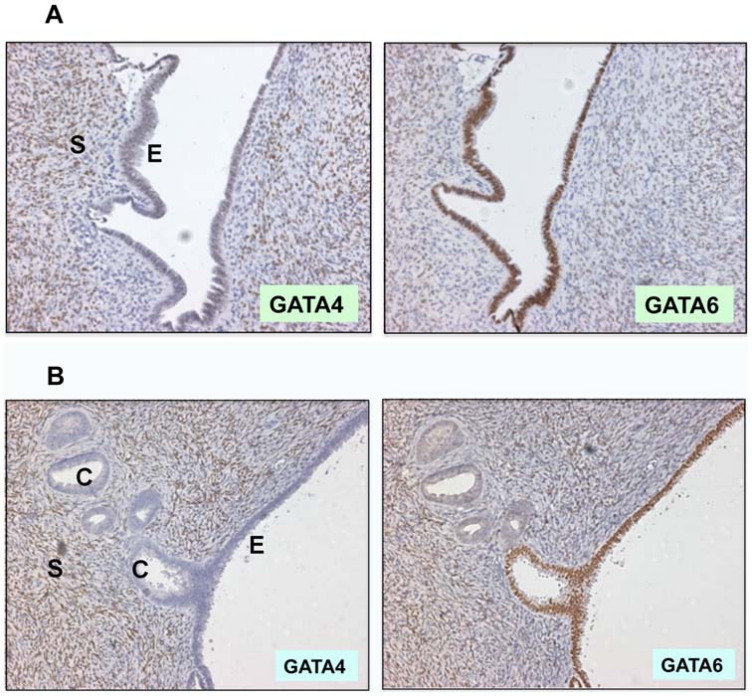
Loss of GATA4 and GATA6 in pre-malignant lesions. Immunostaining of GATA4 and GATA6 in morphologically normal ovarian surface and/or cyst epithelia found in ovarian carcinomas. A. An ovarian surface epithelium is positive for GATA6 and negative for GATA4. Positive GATA4 staining in stroma is noted. B. An ovarian surface epithelium is positive for GATA6 and negative for GATA4. Both GATA4 and GATA6 are lost in cyst epithelial cells adjacent to the surface. 200× magnification. E, epithelium; C, cyst; S, stroma.

Previously, microscopic carcinomas and pre-neoplastic morphological changes were observed in prophylactic oophorectomies, showing the usefulness of these tissues in studying the initiation of ovarian epithelial cancer [37]. In the current analysis, the 26 cases of ovarian tissues from prophylactic oophorectomies were found to contain no microscopic tumors, but did exhibit morphologically altered lesions derived from ovarian surface or cyst epithelia [36]. In some overt ovarian carcinomas, morphologically normal monolayers of epithelial cells can be found adjacent to malignant cells. Just because of the physical proximity, these morphological normal epithelial cells may share some features with the neoplastic cells. By immunostaining, we found that in the majority of the cases GATA4 is absent and GATA6 is positive in the single layered, morphologically benign lesions, as shown by an example (Figure 2A). In a second example, ovarian epithelial cells lining the surface are positive for GATA6 and negative for GATA4, and the epithelial cysts beneath the surface lost both GATA4 and GATA6 expression (Figure 2B).

Each slides of the 26 samples contains variable numbers of lesions with variable degrees of morphological changes. We have examined about 80 of these lesions to make several general descriptions: GATA4 is negative in about 80% of surface epithelia, and GATA4 is absent in nearly all obvious lesions and also about half the morphologically normal monolayers. GATA6 is generally present (70%) in the epithelial lesions, and loss of GATA6 correlates with neoplastic morphological changes (multiple epithelial layering) in about 90% of the cases. Thus, GATA4 loss often precedes GATA6 loss, and loss of GATA4 and GATA6 occurs prior to malignant transformation of ovarian surface epithelial cells.

### Loss of GATA4 and/or GATA6 closely correlates with epithelial morphological transformation

**Figure 3:**
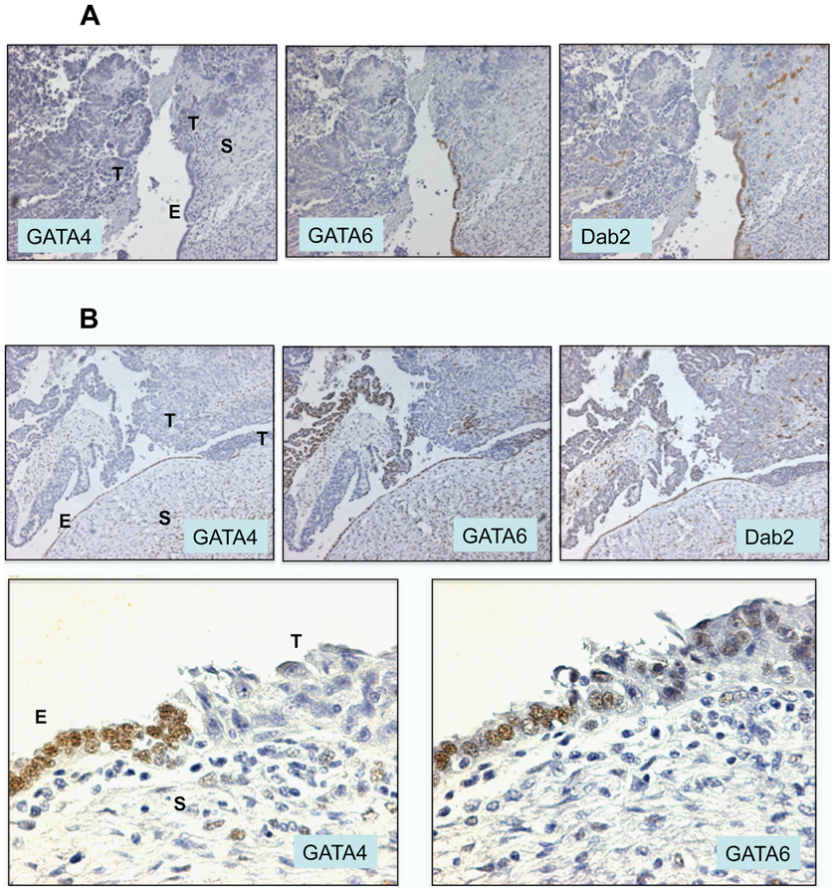
Loss of GATA4 and/or GATA6 closely correlates with epithelial morphological transformation. Examples of human ovarian cancer stained for GATA4, GATA6, and Dab2 in adjacent tumor sections. A. An ovarian surface epithelium that consists of a morphological normal surface epithelium (arrow head) contiguously linking malignant cells (arrow) that have either invaded into stroma or expanded into areas around the ovary. GATA4 is negative in both morphological normal or neoplastic epithelial cells. The epithelial cells in the morphological normal epithelium are positive for GATA6 and Dab2, and the malignant cells are negative for GATA6 and Dab2. Some scattered cells in the stroma are Dab2 positive but negative for GATA6. These cells are macrophages in which Dab2 expression is not GATA6-dependent. B. In this ovarian carcinoma, the epithelial cells in the morphological normal epithelium are positive for GATA4, GATA6, and Dab2, and the adjacent malignant cells are negative for GATA4, GATA6, and Dab2. The images were taken under a microscope with a 100× magnification. The stainings for GATA4 and GATA6 in the transition in panel B are shown in a higher magnification (400×) at the lower panels. E, epithelium; C, cyst; S, stroma; T, tumor.

Over the years, we assembled a collection of 12 ovarian carcinomas that contain lesions of contiguous epithelia linking morphologically benign epithelial cells to overt cancer cells [35]. Such lesions have also been described previously for mucinous and serous ovarian cystadenocarcinomas [38]. We reasoned that the presence of a transition from benign to malignant may be useful to determine the involvement of candidate genes in ovarian epithelial tumorigenesis. Indeed, in all 12 cases of benign to neoplastic transitions analyzed, loss of GATA4 and/or GATA6 closely associates with neoplastic morphological transformation. In the first example (Figure 3A), GATA4 is already absent in the benign monolayer; GATA6 is positive in the monolayer cells but is lost in the adjacent cancer cells. Loss of GATA6 closely correlates with the loss of its transcription target Dab2 (Figure 3A). In a second example, GATA4 and GATA6 expression are lost simultaneously, closely associated with loss of Dab2 and morphological transformation (Figure 3B). The enlarged benign to neoplastic transition site (Figure 3B, lower panels) shows that GATA4 is abruptly lost, and GATA6 is greatly reduced on the transition.

Thus, the loss of GATA6 expression correlates closely with neoplastic transformation of the ovarian surface epithelium, as observed in contiguous ovarian surface epithelium connecting morphologically normal to malignant cells (Figure 3). In previous studies [18], [35], the ovarian surface epithelium immediately adjacent to tumor areas was considered to be pre-neoplastic. These pre-neoplastic lesions often lack an intact basement membrane as indicated by the absence of collagen IV and laminin staining, as well as the loss of Dab2 expression [35]. In the morphologically normal epithelia immediately adjacent to tumor areas, both GATA6 and Dab2 are positive. However, the contiguously connected neoplastic cells are negative for both GATA6 and Dab2 staining. The neoplastic cells in the transformed epithelium that is immediately connected to the morphologically normal ovarian surface epithelial cells are likely most similar to the normal, and thus are considered early tumor cells. Such analysis suggests the possibility that the loss of GATA6 is one of the causative factors of ovarian epithelial transformation.

The observations from human ovarian cancer tissues suggests that loss of GATA4 expression often precedes the loss of GATA6, and the loss of GATA6, correlated with the loss of Dab2, associates with epithelial morphological transformation.

### Reduction of GATA factor expression upon passaging of ovarian surface epithelial cells in primary cultures

Over the last 5 years in the studies of GATA factors in ovarian surface epithelial and culture cells, we found that GATA4 is absent in most ovarian cancer cell lines and also SV-40-transfected, “immortalized” ovarian surface epithelial cells [17], [18]. Additionally, GATA4 expression was variable in primary cultures of human ovarian surface epithelial cells. Thus, we speculate that the expression of GATA4 is not stable in ovarian surface epithelial cells and may gradually reduce over time.

To test this idea, we carefully followed the passages of primary ovarian surface epithelial cells in culture overtime and monitored the expression of GATA4, GATA6, and Dab2 genes. The ovarian surface epithelial cells were harvested from ovaries from prophylactic oophorectomies as described previously [17], [18]. The tissues were examined and were determined to be morphologically normal and void of microscopic tumors. The cell doubling time was about two week initially and shortened to one week to 5 days in later passages. We have carried out the passages of a HOSE cell preparation (ROE 1005816) to passage 10 over a two-month period and the cells were still highly proliferative at the end. Typically, the cells can grow in culture for about 20 passages over a 6-month period before undergoing replicative senescence. At each passage, half of the cells were harvested and used for expression analysis, and the other half was maintained continuously in culture.

**Figure 4:**
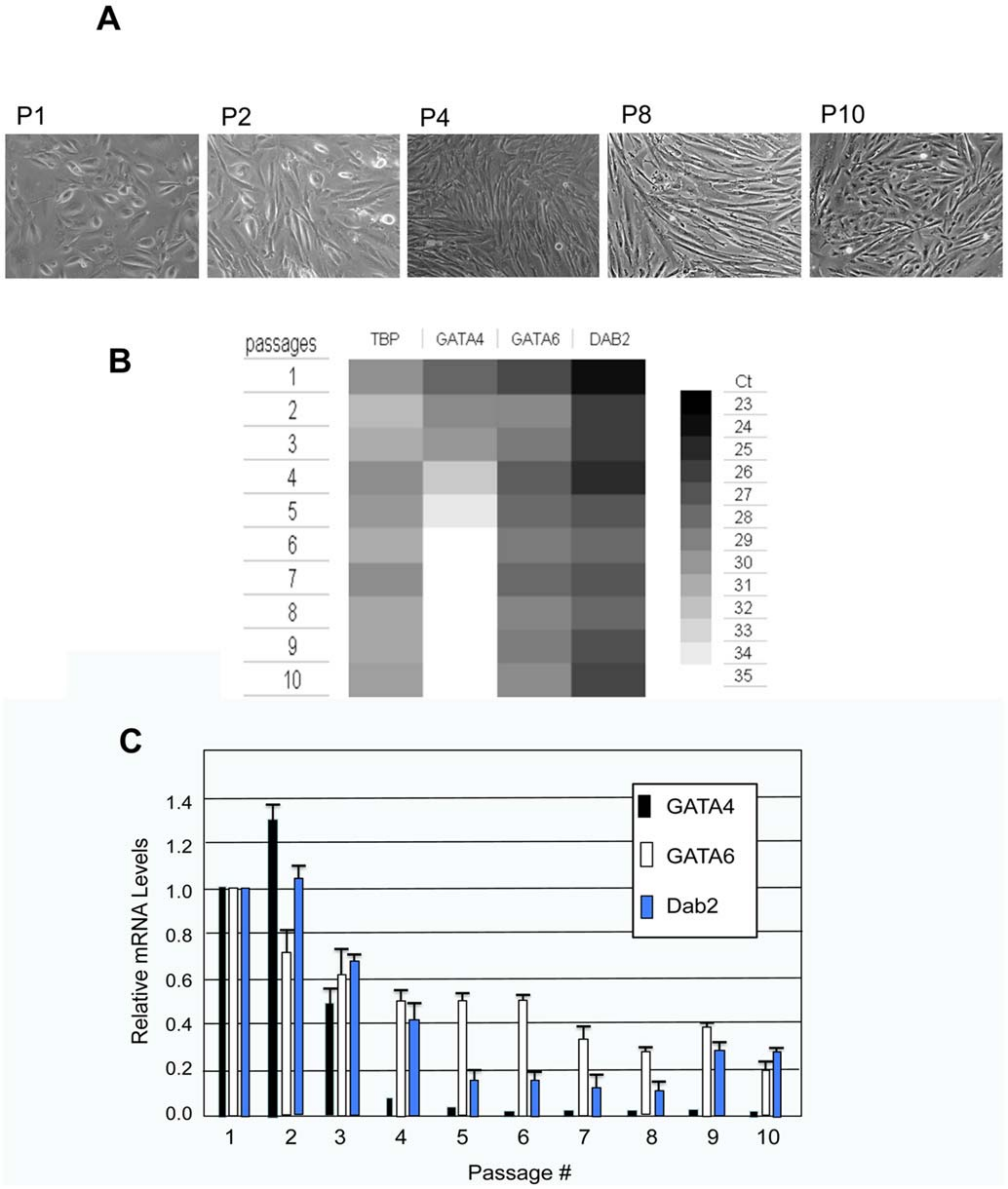
Reduction of GATA4 and GATA6 expression upon culturing of ovarian surface epithelial cells. Human ovarian surface epithelial cells were isolated from morphologically normal human ovaries obtained from oophorectomy surgery. The cells were passaged 10 times over a period of 3 months, and cells were harvested from each passage for mRNA analysis. A. Morphology of the cells in passage #1, 2, 4, 6, 8, 10. B. The total RNA were used for quantitative real-time RT-PCR to assay mRNA levels. The results are presented as a “heat-map”. C. The amount of mRNA relative to passage 1 was quantitated and presented as a bar graph.

First, the changes in the morphology of the ovarian surface epithelial cells were obvious. Initially, the cells appeared to be round and flat, with a “cobblestone” morphology, and increasingly the cells became elongated and spindle-like, and the most dramatic changes was observed at passage 4 (Figure 4A). GATA4 mRNA levels were found to reduced rapidly, and became undetectable by passage 4 (Figure 4B,C). The expression of GATA6 and its transcription target Dab2, reduced gradually over time, and the mRNA levels of both genes were 20–30% of the original cells (Figure 4C). As a control, house-keeping gene TBP (TATA box binding protein, accession number NM_003194) expression level had insignificant changes over the course of the culture, and the variation probably reflects the range of error of the assay.

Thus, all GATA4, GATA6, and Dab2 mRNA levels are reduced upon passaging of the primary ovarian surface epithelial cells in culture. The loss of GATA4 precedes the loss of GATA6 (and Dab2), similar to the observation in the pre-neoplastic ovarian epithelia adjacent to ovarian carcinomas, where GATA4 is often lost prior to GATA6. Previously, the loss of GATA4 and GATA6 in ovarian cancer and epithelial cells was demonstrated to be by the mechanism of hypoacetylation of histones H3 and H4 and loss of histone H3/lysine K4 trimethylation at their promoters [18]. Gene expression is determined by the pattern of associated histone modification, known as the histone codes [39]. Loss of histone acetylation leads to a more compact chromatin conformation and loss of transcriptional activity [40], and histone H3/lysine K4 methylation associates with active genes [41], [42]. The readily loss of GATA4 and GATA6 expression suggests the instability of the epigenetic states/chromatin conformation, consistent with the early loss of GATA factors in ovarian tumorigenesis.

### Alteration of chromatin modification at GATA4 locus associated with loss of expression upon passaging of ovarian surface epithelial cells in primary cultures

**Figure 5:**
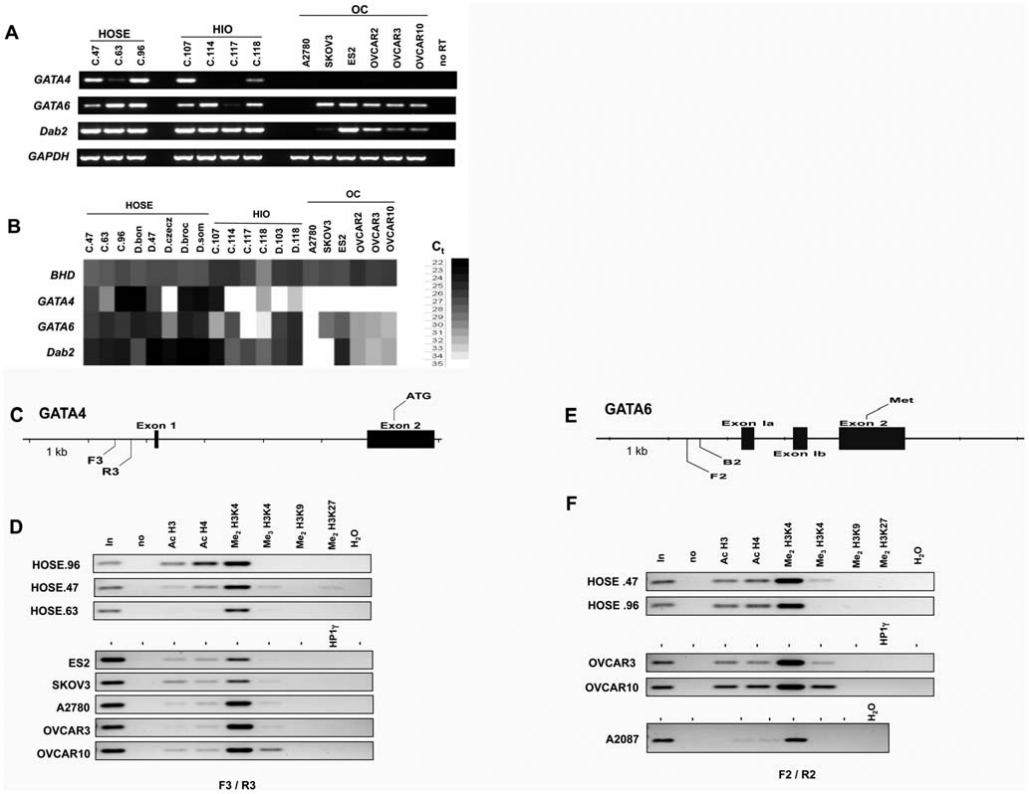
Alterations of histone modifications at GATA4 locus in culturing of ovarian surface epithelial cells. Human ovarian surface epithelial cells were isolated from morphologically normal human ovaries obtained from oophorectomy surgery. Several HIO lines were also established by transfecting the primary cells with SV-40 T-antigen to prolong the life span in cultures. Total RNA isolated from a collection of primary HOSE, HIO, and cancer cells were used for: A. Semi-quantitative RT-PCR, and B. Quantitative real-time RT-PCR, to assay mRNA levels for GATA4, GATA6, Dab2, and GAPDH (semi-quantitative RT-PCR) or BHD (real-time RT-PCR) as loading controls. Sufficient RNA were obtained from HOSE.96 (or C.96), passage #3; HOSE.47 cells, passage #5; and HOSE.63 cells, passage # 12; for CHIP assay. C. The location of primers F3 and R3 at GATA4 gene for PCR in the ChIP assay is shown. D. Lysates from HOSE.96, HOSE.47, HOSE.63, ES2, SKOV3, A2780, OVCAR3, and OVCAR10 were prepared for CHIP assay. Chromatin precipitations by antibodies to Ac H3, Ac H4, Me_2_H3K4, Me_3_H3K4, Me_2_H3K9, Me_2_H3K27, and HPγ1 were used to amplify by PCR using primers F3 and R3. The input signal (“in”, 2% of total chromatins used for immunoprecipitation), no antibody control (“no”), and no template control (“H_2_O”) were included. The bright field images of the semi-quantitative PCR products are shown. E. The location of primers F2 and B2 at GATA6 gene for PCR in the CHIP assay is shown. F. Lysate from HOSE.96, HOSE.47, A2780, OVCAR3, and OVCAR10 were prepared for CHIP assay. The bright field images of the semiquantitative PCR products are shown.

To determine the cause of the reduction of GATA factor expression in primary cultures, we first determine the expression in a large panel of ovarian surface epithelial cells and cancer cells. Total RNA was isolated from 8 preparations of primary HOSE cells (from 7 persons, here, C.47 and D.47 were split from the same HOSE preparations but were cultured by different lab staff), 6 HIO lines which are HOSE cells transfected with SV40-T-antigent to prolong culturing life span, and 6 lines of ovarian cancer cells (Figure 5). The RNA was subjected to semi-quantitative RT-PCR (Figure 5A) and real-time RT-PCR (Figure 5B) to determine mRNA levels for GATA4, GATA6, Dab2, and GAPDH or BHD as loading controls. GATA4 expression is reduced in some HOSE preparations and HIO lines, and is absent in all cancer lines as determined by both semi-quantitative RT-PCR and real-time RT-PCR (Figure 5A,B). Reduction of GATA6 and Dab2 expression in HIO and cancer lines is more apparent by real-time RT-PCR (Figure 5B).

We were able to obtain a sufficient amount of 3 primary HOSE cells (HOSE.96, HOSE.47, and HOSE.63) for further study of changes in chromatin modification of the GATA4 and GATA6 locus by ChIP assay during passaging of the cells in cultures. The expression levels of GATA4 inversely correlate with passage number of the cells: HOSE.96 (or C.96) cells, passage #3, have high GATA4 expression; HOSE.47 cells, passage #5, have a lower GATA4 level; and HOSE.63 cells, passage # 12, have a much reduced expression level (Figure 5B).

Following the previously established protocol for ChIP assay for GATA4 and GATA6 genes [18], we found that, indeed, at a site immediately up-stream of the GATA4 Exon 1 (Figure 5C), the reduction of GATA4 expression levels correlates with the loss of acetylation of Histone H3 and H4 (Figure 5D). No significant changes were seen for di and tri methylation of H3K4, markers for active chromatin conformation [41], [42]. However, dimethylation of histone H3 at lysine 9 and 27 (Me_2_H3K9, Me_2_H3K27) and HP1 is absent, suggesting the silenced GATA4 gene is not being incorporated into heterochromatin [43].

Beside the reduced acetylation of histone H3 and H4 in A2780 cells, the patterns of histone modification at the GATA6 locus (Figure 5E) is similar between HOSE cells and cancer lines OVCAR3 and OVCAR10 (Figure 5F), suggesting the alteration of histone modification at GATA6 locus is not a general event in ovarian epithelial and cancer cells.

### Ovarian tumor prone phenotype of GATA6 heterogeneous mice

**Figure 6:**
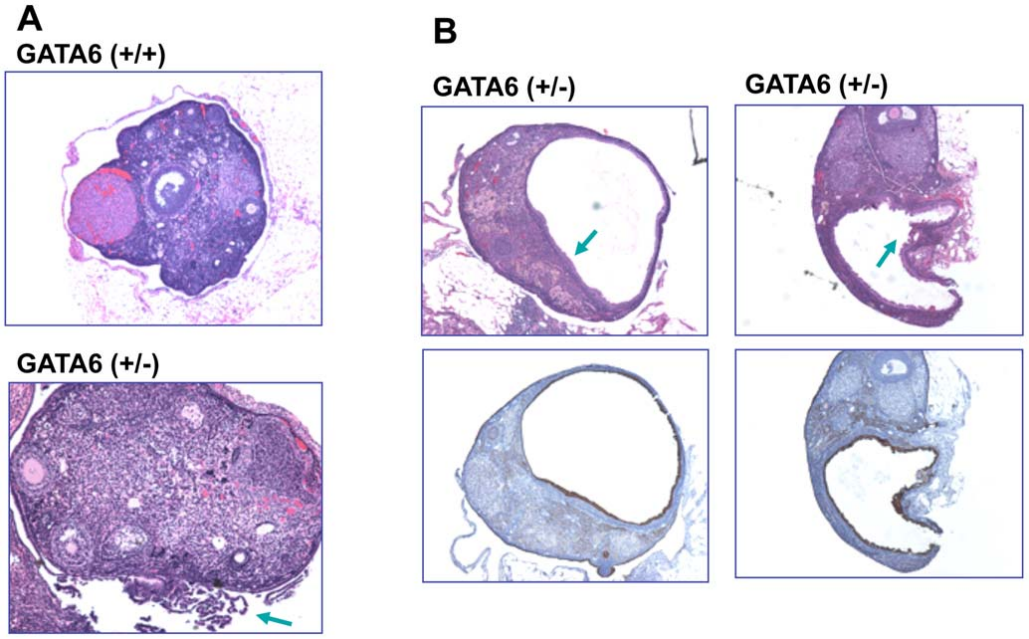
Ovarian morphology of GATA6 heterogeneous mice. The ovarian morphology of GATA6 (+/−) and wildtype littermates of about one year of age was compared. Cytokeratin staining was used as a marker for epithelial cells. A. A typical example of a wildtype ovary shows numerous follicles in various developmental stages enclosed inside a layer of surface epithelium. A representative GATA6 (+/−) ovary shows the presence of outgrowth of epithelial cells from the surface (arrow). B. A large portion of GATA6 (+/−) ovaries also exhibits large cysts inside the ovaries. The cells lining the cysts are cytokeratin positive (lower panels). All images were taken under a microscope with a 100× magnification.

Finding a close correlation between the loss of GATA6 and epithelial transformation prompted us to speculate that the loss of GATA6 may be causative in the transformation of ovarian surface epithelial cells. The above observations also suggest that the loss of GATA6 may be more critical than the loss of GATA4 in promoting ovarian epithelial transformation. To determine the consequence for the loss of GATA6 in ovarian surface epithelia, we investigated the ovarian phenotype of GATA6 (+/−) mice, since the homozygous mice are embryonic lethal [24], [25]. We have obtained and maintained a colony of GATA6 knockout mice over the last 2 years. Ovarian tissues were harvested from the mice at around one year of age, and morphology of ovaries from littermates of GATA6 (+/−) and wildtype were compared. Strikingly, all ovaries of GATA6 (+/−) genotype exhibited some abnormalities (Table 2): Some ovaries have extensive proliferative lesions of the surface epithelia (Figure 6A), and the others have large ovarian cysts that are positive for cytokeratin (Figure 6B). The ovarian phenotype resembles those of *dab2* (+/−) mice [32], consistent with Dab2 as a GATA6 transcription target and mediator [27].

**Table 2:** Pathology report in GATA6 Heterozygous mouse ovaries.

Path. No.	Age (Month)	Inclusion Cyst	Papillomatosis	Epithelial Hyperplasia	notes
06-4201	10	-	+	+	
06-4418	7	-	-	-	Uterine epithelial hyperplasia
06-4222A	15.5 M	-	-	-	
06-4223A	15.5	-	-	-	
06-4224A	15.5	+	-	+	Ovarian cystadenoma, uterine cystic change
06-4225A	15.5	-	-	-	Lymphoma in ovary
06-4226A	15.5	-	+	-	Ovary atrophy
06-4227A	14.5	-	-	-	
06-4228A	14.5	-	-	-	Ovarian cystadenoma
06-4229B	16	-	-	-	
06-4230	16	-	+	-	
06-4231	12	-	-	-	
06-4232	11.5	-	-	-	
06-4446	14	-	-	-	
06-4447	15.5	-	-	-	Lymphoma in ovary
06-4448	18	-	-	-	Lymphoma in ovary
06-4449	15.5	-	-	-	
06-4450	18	+	+	-	
06-4452	15.5	-	-	+	
06-4455	13.5	-	-	-	
06-4456	13.5	+	-	+	
06-4458	13.5	+	-	+	
06-4459	13	-	-	-	
06-4460	13	+	-	-	
06-4461	13	-	-	-	
06-4462	13	-	-	-	
06-4483	17	-	-	-	-
06-4892	24	+	-	-	-
06-4893	16.5	+	-	-	-
06-4899	15.5	-	-	-	-
06-4901	16	-	-	-	-
06-4902	16	-	-	-	-
06-4903	16	-	-	-	-
07-5780	12	+	-	-	-
07-5781	15	-	+	-	-
08-4142	12	-	-	-	-
08-4143	13	+	-	-	-
08-4144	13	-	-	-	-
08-4145	13	-	-	-	-
08-4146	13	+	-	-	-
08-4147	13	-	-	-	-
08-4148	13	-	-	-	-
08-4149	13	-	-	-	-
08-4150	13	+	-	-	-
08-4151	13	-	-	-	
08-4152	13	+	-	+ +	Extensive ovarian surface hyperplasia
08-4154	13	-	-	-	

A total of 47 GATA6 (+/−) mice were analyzed for ovarian phenotype. The majority of mice were analyzed between the age of 12–18 months. Ovarian tissues were harvested and subjected to histological analysis. More than 20 wildtype littermates were also analyzed as controls. The 47 GATA6 (+/−) mice exhibited the presence of ovarian inclusion cysts in 12 (26%), surface papillomatosis in 5 (11%), and hyperplasia of surface epithelial cells in 6 (13%). Only one in the 20 wildtype mice of same age range showed mild surface papillomatosis.

Thus, this result suggests GATA6 deficiency influences ovarian surface epithelia, possibly promoting transformation though tissue specific deletion of GATA6 in ovarian surface epithelial cells will be a better test, when GATA6 conditional knockout mice become available.

## Discussion

This study examined the expression of GATA 4 and GATA6 in ovarian surface epithelial cells and carcinomas in detail, and found that the loss of GATA4 is more wide spread and often precedes GATA6 in the transformation of ovarian surface epithelia. The loss of GATA6, however, correlates closely with the morphological transformation of the epithelia. The readily reduction of GATA4 and GATA6 expression upon culturing of the ovarian surface epithelial cells suggests the instability of the epigenetic state/chromatin conformation of GATA4 and GATA6 locus, consistent with the early loss in ovarian tumorigenesis. The early loss of GATA4 and GATA6 and the close correlation with the neoplastic transformation of ovarian surface epithelia suggests a causative role, which is supported by the prevalence of ovarian epithelial lesions in GATA6 heterozygous deficient mice [32].

In contrast to other histological subtypes, mucinous ovarian carcinomas generally are positive for GATA4 and GATA6 expression. Indeed, the morphology of mucinous ovarian carcinomas is very unique in that the cancer cells appear to retain apical polarity and often exist as monolayer epithelia.

The quickly loss of GATA4 in passaging of primary cells in culture is intriguing. The loss of GATA4 expression in passaging of primary cells in culture associates with loss of H3 and H4 histone acetylation at the GATA4 promoter locus. The loss of GATA4 in cancer cell lines appears also mediated by the reduction of H3 and H4 histone acetylation of the promoter locus. Thus, it may be speculated that chromatin changes led to the loss of GATA4 occurs at the very early stage of ovarian epithelial transformation. Loss of GATA4 itself may not present any phenotype. However, the loss of GATA4 may lead to the destabilization of the gene expression profile of the differentiated ovarian surface epithelial cells. Further changes may lead to loss or reduction of GATA6 expression. The loss of GATA6, and subsequently its transcription target Dab2, may be causative for morphological transformation of ovarian surface epithelia.

The finding of the loss of GATA4 at the very early, non-phenotypic stage preceding ovarian surface epithelial tumorigenesis may provide insight to the risk factors and initial step in the development of ovarian cancer. The ovarian phenotypes of heterozygous GATA6 knockout mice suggest a causative role for the loss of GATA factors in ovarian tumorigenesis. Further evidence may be obtained by examining conditional GATA4 and GATA6 knockout mice that can bypass the requirement of GATA factors in embryonic development.

## Materials and Methods

### Ovarian Tissues and Tumor Specimens and Immunohistochemistry

Cancerous and benign ovarian specimens were obtained from patients who underwent surgical resection at Fox Chase Cancer Center. The 3 ovarian tumor tissue microarrays (TMAs) and 20 prophylactic oophorectomies were provided by the Tumor Bank Facility of Fox Chase Cancer Center. We also used a collection of 38 separate cases of archived ovarian tumor tissue blocks. The tumors were histologically classified according to the World Heath Organization (WHO) classification and the surgical stages were determined according to the classification of International Federation of Gynecology and Obstetrics (FIGO). Prior to surgeries, a blanket informed consent was obtained from the patients in written for the agreement of general laboratory study of the tissues following pathological diagnosis. The current study of human ovarian tissues and tumor samples was reviewed and approved by the Fox Chase Cancer Center IRB (Institutional Review Broad). The tissues were coded and disconnected from patient names and personal information prior to collection by the Institutional Tumor Bank, and the study was considered exempted by the IRB.

Immunostaining was performed using the mouse DAKO Envision TM^+^ System and the Peroxidase (DAB) Kit (Dako Corporation, Carpinteria, CA, USA) as previously described [17], [36]. The antibodies used include: anti-GATA6, anti-GATA4, anti-Dab2/p96 (1∶400 dilution, clone 52, BD Transduction Laboratories, Lexington, KY). Negative controls were prepared by replacing the primary antibody with mouse non-immunized IgG. Evaluation of the staining intensity and characteristics were performed independently by two pathologists in a blinded manner. Tumors with more than 10% of tumor cells or stromal area showing strong to moderate staining are scored as positive.

### Cell Culture

The panel of human “immortalized” ovarian (HIO) epithelial cell lines [17], two preparations of primary human ovarian surface epithelial cells [36], and ovarian cancer cell lines were analyzed. Ovarian cancer cells OVCAR2, -3, -4, -5, -8, and -10 were collected in Dr. Andrew Godwin's lab and were used previously [17]. Other ovarian cancer cells including A2780, ES2, and SKOV3 were purchased from American Type Culture Collection (ATCC), where information about these ovarian cancer cells is available. The primary and HIO- ovarian surface epithelial cells were cultured in medium 199 and MCDB 105 (1∶1) supplemented with 15% FBS, 0.25 U/ml insulin, 2 mM L-glutamine, 100 U/ml penicillin and 100 µg/ml streptomycin.

Primary cells cultured between passages 2-4 were used for experiments. The ovarian cancer cell lines were cultured in RPMI 1640 supplemented with 10% FBS, 2 mM L-glutamine, 100 U/ml penicillin and 100 µg/ml streptomycin. The cultures were maintained in a humidified atmosphere of 95% air/5% CO_2_ at 37°C.

### Chromatin immunoprecipitation and PCR analysis

Chromatin immunoprecipitation (ChIP) was carried out using the ChIP assay kit (Upstate Biotechnology Laboratories) essentially as sugggested by the manufacturer and described in details previously [18]. The procedures for real time quantitative and semiquantitative RT-PCR was also described previously [18].

### GATA6-deficient Mice and tissue analysis

The founder pair of GATA6 knockout mice was a gift from Dr. Edward Morrisey (University of Pennsylvania) [25]. The colonies were maintained by inbreeding in the Animal Facility of Fox Chase Cancer Center following the protocol of published work [25].

## References

[1] Bell DA (2005). Origins and molecular pathology of ovarian cancer.. Mod Pathol.

[2] Scully RE (1995). Pathology of ovarian cancer precursors.. J Cell Biochem Suppl.

[3] Aoki Y, Kawada N, Tanaka K (2000). Early form of ovarian cancer originating in inclusion cysts. A case report.. J Reprod Med.

[4] Feeley KM, Wells M (2001). Precursor lesions of ovarian epithelial malignancy.. Histopathology.

[5] Boyd J (2008). Whence epithelial ovarian carcinoma?. Gynecol Oncol.

[6] Dubeau L (1999). The cell of origin of ovarian epithelial tumors and the ovarian surface epithelium dogma: does the emperor have no clothes?. Gynecol Oncol.

[7] Lee Y, Miron A, Drapkin R, Nucci MR, Medeiros F (2007). A candidate precursor to serous carcinoma that originates in the distal fallopian tube.. J Pathol.

[8] Seidman JD, Russell P, Kurman RJ, Kurman RJ (2002). Surface epithelial tumors of the ovary.. Blaustein's Pathology of the Female Genital Tract.

[9] Richardson GS, Scully RE, Nikrui N, Nelson JH (1985). Common epithelial cancer of the ovary (2).. N Engl J Med.

[10] Hanahan D, Weinberg RA (2000). The hallmarks of cancer.. Cell.

[11] Berchuck A, Kohler MF, Marks JR, Wiseman R, Boyd J, Bast RC (1994). The p53 tumor suppressor gene frequently is altered in gynecologic cancers.. Am J Obstet Gynecol.

[12] Boyd J, Hamilton TC, Berchuck A, Hoskins WJ, Perez CA, Young RC (2000). Oncogenes and tumor-suppressor genes.. Principles and Practice of Gynecologic Oncology (Third ed.).

[13] Ozols RF, Bookman MA, Connolly DC, Daly MB, Godwin AK, Schilder RJ, Xu XX, Hamilton TC (2004). Focus on epithelial ovarian cancer.. Cancer Cell.

[14] Fusenig NE, Breitkreutz D, Boukamp P, Tomakidi P, Stark HJ (1995). Differentiation and tumor progression.. Recent Results Cancer Res.

[15] Lynch RG (1995). Differentiation and cancer: the conditional autonomy of phenotype.. Proc Natl Acad Sci USA.

[16] Rheinwald JG, Beckett MA (1980). Defective terminal differentiation in culture as a consistent and selectable character of malignant human keratinocytes.. Cell.

[17] Capo-chichi CD, Roland IH, Vanderveer L, Bao R, Yamagata T (2003). Anomalous expression of epithelial differentiation-determining GATA factors in ovarian tumorigenesis.. Cancer Res.

[18] Caslini C, Capo-Chichi CD, Roland IH, Nicolas E, Yeung AT, Xu XX (2006). Histone modifications silence the GATA transcription factor genes in ovarian cancer.. Oncogene.

[19] Molkentin JD (2000). The zinc finger-containing transcription factors GATA-4, -5, and -6. Ubiquitously expressed regulators of tissue-specific gene expression.. J Biol Chem.

[20] Kuo CT, Morrisey EE, Anandappa R, Sigrist K, Lu MM (1997). GATA4 transcription factor is required for ventral morphogenesis and heart tube formation.. Genes Dev.

[21] Morrisey EE, Ip HS, Lu MM, Parmacek MS (1996). GATA-6: a zinc finger transcription factor that is expressed in multiple cell lineages derived from lateral mesoderm.. Dev Biol.

[22] Capo-Chichi CD, Rula ME, Smedberg JL, Vanderveer L, Parmacek MS (2005). Perception of differentiation cues by GATA factors in primitive endoderm lineage determination of mouse embryonic stem cells.. Dev Biol.

[23] Koutsourakis M, Langeveld A, Patient R, Beddington R, Grosveld F (1999). The transcription factor GATA6 is essential for early extraembryonic development.. Development.

[24] Cai KQ, Capo-Chichi CD, Rula ME, Yang DH, Xu XX (2008). Dynamic GATA6 expression in primitive endoderm formation and maturation in early mouse embryogenesis.. Dev Dyn.

[25] Morrisey EE, Tang Z, Sigrist K, Lu MM, Jiang F, Ip HS, Parmacek MS (1998). GATA6 regulates HNF4 and is required for differentiation of visceral endoderm in the mouse embryo.. Genes Dev.

[26] Wakana K, Akiyama Y, Aso T, Yuasa Y (2006). Involvement of GATA-4/-5 transcription factors in ovarian carcinogenesis.. Cancer Lett.

[27] Morrisey EE, Musco S, Chen MY, Lu MM, Leiden JM, Parmacek MS (2000). The gene encoding the mitogen-responsive phosphoprotein Dab2 is differentially regulated by GATA-6 and GATA-4 in the visceral endoderm.. J Biol Chem.

[28] Fazili Z, Sun W, Mittelstaedt S, Cohen C, Xu XX (1999). Disabled-2 inactivation is an early step in ovarian tumorigenicity.. Oncogene.

[29] Yang DH, Cai KQ, Roland IH, Smith ER, Xu XX (2007). Disabled-2 is an epithelial surface positioning gene.. J Biol Chem.

[30] Yang DH, Smith ER, Roland IH, Sheng Z, He J, Martin WD, Hamilton TC, Lambeth JD, Xu XX (2002). Disabled-2 is essential for endodermal cell positioning and structure formation during mouse embryogenesis.. Dev Biol.

[31] Morris SM, Tallquist MD, Rock CO, Cooper JA (2002). Dual roles for the Dab2 adaptor protein in embryonic development and kidney transport.. EMBO J.

[32] Yang DH, Fazili Z, Smith ER, Cai KQ, Klein-Szanto A (2006). Disabled-2 heterozygous mice are predisposed to endometrial and ovarian tumorigenesis and exhibit sex-biased embryonic lethality in a p53 null background.. Am J Pathol.

[33] Mishra SK, Keyel PA, Hawryluk MJ, Agostinelli NR, Watkins SC, Traub LM (2002). Disabled-2 exhibits the properties of a cargo-selective endocytic clathrin adaptor.. EMBO J.

[34] Sheng Z, Sun W, Smith E, Cohen C, Sheng Z, Xu XX (2000). Restoration of positioning control following Disabled-2 expression in ovarian and breast tumor cells.. Oncogene;.

[35] Yang DH, Smith ER, Cohen C, Wu H, Patriotis C (2002). Molecular events associated with dysplastic morphologic transformation and initiation of ovarian tumorigenicity.. Cancer.

[36] Cai KQ, Klein-Szanto A, Karthik D, Edelson M, Daly MB (2006). Age-dependent morphological alterations of human ovaries from populations with and without BRCA mutations.. Gyn Oncology.

[37] Salazar H, Godwin AK, Daly MB, Laub PB, Hogan WM (1996). Microscopic benign and invasive malignant neoplasms and a cancer-prone phenotype in prophylactic oophorectomies.. J Natl Cancer Inst.

[38] Puls LE, Powell DE, DePriest PD, Gallion HH, Hunter JE (1992). Transition from benign to malignant epithelium in mucinous and serous ovarian cystadenocarcinoma.. Gynecol Oncol.

[39] Jenuwein T, Allis CD (2001). Translating the histone code.. Science.

[40] Mutskov V, Felsenfeld G (2004). Silencing of transgene transcription precedes methylation of promoter DNA and histone H3 lysine 9.. EMBO J.

[41] Santos-Rosa H, Schneider R, Bannister AJ, Sherriff J, Bernstein BE (2002). Active genes are tri-methylated at K4 of histone H3.. Nature.

[42] Schneider R, Bannister AJ, Myers FA, Thorne AW, Crane-Robinson C, Kouzarides T (2004). Histone H3 lysine 4 methylation patterns in higher eukaryotic genes.. Nat Cell Biol.

[43] Lachner M, O'Carroll D, Rea S, Mechtler K, Jenuwein T (2001). Methylation of histone H3 lysine 9 creates a binding site for HP1 proteins.. Nature.

